# Visuo-perceptual capabilities predict sensitivity for coinciding auditory and visual transients in multi-element displays

**DOI:** 10.1371/journal.pone.0183723

**Published:** 2017-09-13

**Authors:** Hauke S. Meyerhoff, Nina A. Gehrer

**Affiliations:** 1 Leibniz-Institut für Wissensmedien, Tübingen, Germany; 2 Department of Psychology, University of Tübingen, Tübingen, Germany; University of Bath, UNITED KINGDOM

## Abstract

In order to obtain a coherent representation of the outside world, auditory and visual information are integrated during human information processing. There is remarkable variance among observers in the capability to integrate auditory and visual information. Here, we propose that visuo-perceptual capabilities predict detection performance for audiovisually coinciding transients in multi-element displays due to severe capacity limitations in audiovisual integration. In the reported experiment, we employed an individual differences approach in order to investigate this hypothesis. Therefore, we measured performance in a useful-field-of-view task that captures detection performance for briefly presented stimuli across a large perceptual field. Furthermore, we measured sensitivity for visual direction changes that coincide with tones within the same participants. Our results show that individual differences in visuo-perceptual capabilities predicted sensitivity for the presence of audiovisually synchronous events among competing visual stimuli. To ensure that this correlation does not stem from superordinate factors, we also tested performance in an unrelated working memory task. Performance in this task was independent of sensitivity for the presence of audiovisually synchronous events. Our findings strengthen the proposed link between visuo-perceptual capabilities and audiovisual integration. The results also suggest that basic visuo-perceptual capabilities provide the basis for the subsequent integration of auditory and visual information.

## Introduction

At any moment, an overwhelming amount of visual and auditory information arrives at the human sensory system. These different kinds of information need to be integrated into a common representation during perception in order to extract a coherent representation of the outside world [1]. The integration of auditory and visual sensory information facilitates stimulus detection [2] and contributes to the recognition of objects [3], the interpretation of visual information [4], an effective employment of attentional resources [5] and enhances long-term memory performance [6]. The occurrence of audiovisual integration depends on the features of the stimuli themselves such as their temporal synchrony [7,8], their spatial coincidence [9,10], or their semantic congruency [11,12].

Research across different tasks that required either the integration of audiovisual information or responses to audiovisually coinciding events has revealed large individual differences between observers. For instance, audiovisual integration in single-element displays can be predicted from individual differences in the width of the temporal binding window [13]. Further, there is electrophysiological as well as neuroimaging evidence that individual differences in early neural responses to audiovisually coinciding events predict subsequent behavioral performance [14,15]. The rationale of the work reported here was to further investigate the origin of individual differences in sensitivity for audiovisually synchronous events. More specific, we employ an individual differences approach in order to investigate whether visuo-perceptual capabilities predict the ability to discriminate the presence versus absence of audiovisually synchronous events in multi-element displays.

Spatial auditory information has been demonstrated to guide visual attention toward the corresponding spatial location. For instance, McDonald et al. [5] showed that detection performance for briefly presented visual stimuli was more accurate when a preceding auditory cue emerged from the same spatial location than when the tone emerged from the opposite side of the visual field [16,17]. The efficiency in guiding visual attention of auditory as well as audiovisual cues is comparable to purely visual cues [18]. In contrast to purely visual cues, however, audiovisual cues maintain their efficiency in guiding attention even at higher perceptual loads [19,20]. Remarkably, however, coinciding tones do not need to carry spatial information in order to bias visual attention toward the corresponding object locations. This effect–labeled the *pip-and-pop effect—*was first demonstrated by van der Burg et al. [21]. In their study, participants searched for a horizontally or vertically oriented bar among tilted bars in a cluttered display. All bars changed their color within intervals of 900 ms. Whenever the target changed color, no other element switched color 150 ms before or 100 ms after this change [7,22]. In one half of all trials, a brief tone coincided with the color change of the target object, whereas no sounds were present in the remaining half of the trials. In trials with coinciding tones, there was a tremendous increase in search efficiency. In several follow-up experiments, van der Burg et al. [21] attributed this increase in search efficiency to the integration of the coinciding auditory and visual transients which in return increases the saliency of the visual target, thus guiding attention toward the corresponding spatial location [23]; but see also [24].

In a subsequent study, van der Burg et al. [25] used the pip-and-pop paradigm to explore the influence of the attentional window on attentional guidance by coinciding auditory and visual transients. In this line of research, the attentional window refers to the size of the focus of attention which is altered by the demands of the (preceding) experimental task. In the experiments, the participants performed a letter matching task prior to the visual search display. Van der Burg et al. manipulated the size of the letter in order to adapt the attentional window. When the letter covered a large portion of the display, a large attentional window was necessary to identify the letter whereas a substantially smaller attentional window was necessary when the letter covered only a small area in the center of the display. Van der Burg et al. observed a less efficient guidance of visual attention due to audiovisual synchrony when the target object appeared outside the previously induced attentional window. This finding matches research within the visual domain showing that attentional capture arises only within the attentional window [26,27]. For the purpose of the reported experiment, findings such as this one show that visual properties such as the size of the attentional window might affect audio-visual integration.

As briefly outlined above, there is remarkable variance in tasks that rest upon audiovisual integration which can be attributed to individual differences in perceptual capabilities. In a study by Stevenson et al. [13], the temporal precision of audiovisual perception (i.e., the width of the temporal binding window) predicted the susceptibility to audiovisual illusions. Because there were no competing stimuli, selection processes were not relevant for this study. However, two studies including competing stimuli [28,29] reported severe capacity limitations for the detection of audiovisually synchronous events. This finding shows that matching sounds to visual objects is not possible for all visual objects in a display in parallel. In this line of evidence, temporal synchrony between auditory and visual transients might guide attention toward the corresponding candidate objects. Here, we hypothesize that individual differences in visuo-perceptual capabilities predict individual differences in the ability to discriminate the presence of coinciding auditory and visual information from their absence because these capabilities limit the temporal precision of visual processing and therefore affect the integration of auditory and visual information.

In the present study, we investigated the proposed link between visuo-perceptual capabilities and the discriminability of the presence versus absence of audiovisual synchrony. We employed an individual differences approach that aimed at predicting performance in discriminating the presence and absence of audiovisually synchronous events in multi-element displays from visuo-perceptual capabilities. As a proxy for these basic visuo-perceptual processes, we used a variant of the useful-field-of-view task (UFOV) which has been used before to study individual differences in perceptual capabilities [30,31]. In this task, participants are asked to detect very brief visual stimuli that appear in the visual field. As a proxy for the increase in saliency of visual events due to coinciding auditory information, we asked participants to discriminate between trials in which brief tones coincided with the direction changes of one of multiple moving objects and trials in which identical tones were independent of any direction changes of the moving objects (AVS task; see also [32]). In order to control for other potential superordinate influences on both tasks, such as motivation of the participants, we also conducted a color-visual-short-term-memory (CVSTM) task [33,34]. We chose this task because it is a well-established task measuring the capability to memorize items rather than detecting them. Due to their conceptual independence, we expected performance in this task to be independent of UFOV performance as well as performance in the visual search task for audiovisually synchronous direction changes.

## Methods

### Participants

The final sample consisted of 23 students (20 female, 19–29 years) from the University of Tübingen. No participant reported visual or auditory impairments. Additional data from one participant were excluded due to near-chance level performance in both relevant tasks (removing this participant actually lowered the correlation between UFOV and AVS performance). Our research was conducted in accordance with APA standards for ethical treatment of subjects and with the approval of the institutional review board of the Leibniz-Institut für Wissensmedien, Tübingen, Germany. All participants provided (written) informed consent prior to testing.

### Apparatus

All stimuli were presented on a 27” LCD (1920 x 1080 pixels, 60 Hz) monitor controlled by a MacMini. The stimuli were presented with luminance values of 286 cd/m^2^ (white) and 61.5 cd/m^2^ (grey) whereas the background remained at 0.2 cd/m^2^ (black). All tasks were programmed in Python using the PsychoPy libraries [35].

### Tasks and stimuli

#### Useful-field-of-view task (UFOV)

**Fig 1:**
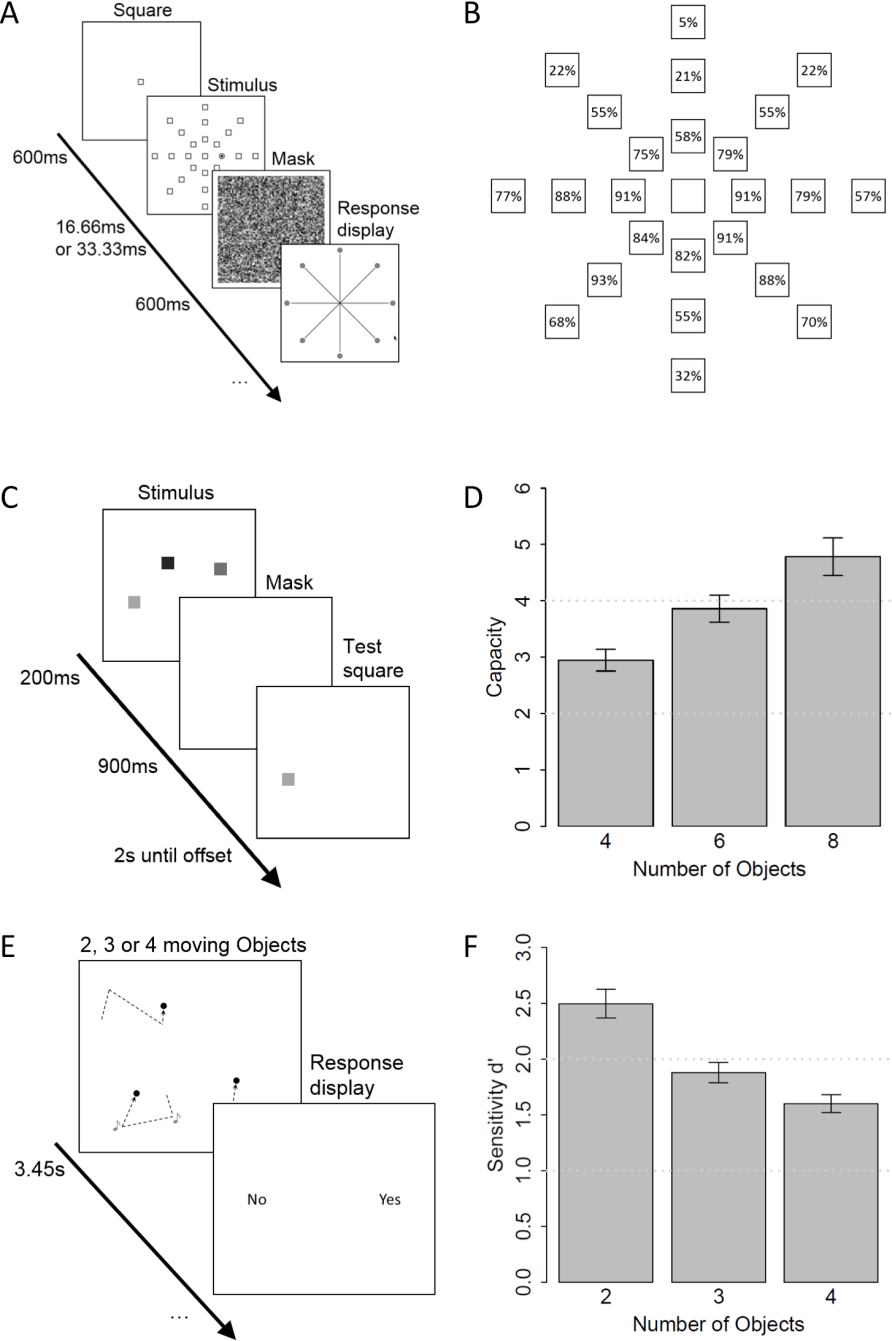
Stimuli and results of the three tasks. A: Stimuli of the useful-field-of-view task (UFOV). In this task, participants reported the location of briefly presented target stimuli. B: Results of the UFOV task. The numbers within the squares indicate the average identification of a target at the corresponding location. For the main analysis, we calculated the average performance of each participant. C: color-visual-short-term-memory task (CVSTM). In this task, participants retrieved location as well as color information from a previously presented display. D: Results of the CVSTM task across different set sizes. For the main analysis, we calculated the average capacity for each participant. E: Illustration of the audiovisual synchrony task (AVS). In this task, participants discriminated between trials in which tones coincided with the visual direction changes of one of the objects and trials in which identical tones were unrelated to any direction change. Presentation duration was constant at 3.45s. F: Results of the AVS task. For the main analysis, we calculated the sensitivity measure d’ for the data collapsed across all set sizes.

In this task, participants were asked to detect stimuli across a large visual field that were masked briefly after their presentation (see Fig 1A). Each trial started with the presentation of an empty white square (3 x 3 deg) against a black background in the center of the screen. After 600 ms, 24 additional squares appeared onscreen. These squares were arranged on eight invisible axes with eccentricities of 10, 20, and 30 degrees of visual angle resulting in a total visual field of 60 deg. One of these squares was replaced by a target circle (3.46 deg in diameter) filled with a smaller filled square (1.5 x 1.5 deg); participants were instructed to detect this target. Each square was probed equally often. In order to avoid ceiling or floor effects, the target display was presented for one frame (16.7 ms) when the target appeared at an eccentricity of 10 deg, but for two frames when the target appeared at the larger eccentricities. Immediately after the presentation of the target display, the entire visual field was masked by a pattern of random pixels ranging from black to white for 600 ms. Following this, a response display appeared and participants selected the axis of the target with a mouse click. The participants were instructed to guess when they did not see the target in the preceding display. Each participant completed five blocks consisting of 24 trials. Prior to the experimental trials, participants completed three practice trials with slower presentation speed in order to become familiar with the task.

(This task has been used in order to measure the size of the useful field of view in previous work. In our study, however, it appears that the task captures the detectability of briefly presented visual stimuli rather than the size of the visual field as individual difference are stable even at the smallest eccentricities.)

#### Color-visual-short-term-memory task (CVSTM)

In this task, participants were asked to recognize location-feature bindings across brief retention intervals (see Fig 1C). In each trial, we presented a 200 ms stimulus display consisting of four, six, or eight colored squares (1 x 1 deg) against a gray background. The colors of the squares were sampled with replacement from the colors black, white, red, blue, yellow, green, cyan, and magenta. All stimuli appeared in the center of the screen within a field of 10 x 8 deg. Immediately after this stimulus interval, there was a blank screen for 900 ms which served as a retention interval. Then a probe appeared at the location of one of the previous stimulus locations. In one half of the trials, this probe had the same color as the object that had previously been at that location. The participants were asked to press the corresponding buttons on a keyboard to indicate whether it matched the previous object at that location. The probe remained onscreen until a response was recorded or for a maximum of two seconds. Responses were nevertheless possible even after the offset of the probe display. Each participant completed three blocks of 40 trials. The number of stimuli increased with every new block (4 vs. 6 vs. 8; in order to maintain comparability to previous research on this task, we inherited this blockwise increase in set size). Prior to testing, the participants completed eight trials with two objects to become familiar with the task.

#### Audiovisual-synchrony task (AVS)

In this task, participants were asked to distinguish between trials in which a brief tone coincided with the direction changes of one out of two, three, or four objects and trials in which the tone did not coincide with any visual direction change (see Fig 1E). The stimuli were white discs (0.6 deg in diameter, ~140 cd/m^2^) that moved within an invisible square (19 x 19 deg) with a constant speed of 4.5 deg per second. The angular difference between old and new directions of motion was sampled from a uniform distribution with a minimum of 60 deg and a maximum of 120 deg. Importantly, no object changed its direction of motion 150 ms before or after the direction change of another object. Additionally, no object changed its direction of motion within the first 250 ms of each trial and there were at least 250 ms between two direction changes of the same object. In order to avoid spatial overlaps between distinct objects, each object was randomly assigned to one of the four quarters and remained within this quarter for the entire trial. In trials with less than four objects, the remaining quarters remained empty. In order to also avoid collision events, the objects were prevented from moving within the area of 0.2 deg around the centerlines of the invisible quarters.

In each trial, each visual object underwent three visual direction changes (one within each interval of 1.15 seconds) and there were also three tones (500 Hz, 50 ms, ~60 dB). In target-present trials, the tones coincided with the direction changes of one of the objects, whereas in target-absent trials, the tones coincided with the direction changes of an additional (invisible) object that followed the same constraints as the remaining four objects. We used a fixed presentation duration in order to avoid speed-accuracy trade-offs. After the full event, participants registered their response by pressing the corresponding button on a keyboard. Each participant completed 15 blocks of 24 trials. Prior to testing, the participants completed six practice trials.

### Procedure

All participants completed all tasks starting with the UFOV task. In this task, the fixed viewing distance (ensured by a chin rest) was 28 cm in order to establish the larger visual field. After completion, the participants were seated in front of a second, identical experimental setup with a restricted viewing distance of 60 cm where they completed the CVSTM task before ending the experiment with the AVS task. In all tasks, the stimuli were generated offline and all participants completed the same trials in the same order. The entire experiment took approximately 60 minutes to complete.

## Results

In line with the proposed link between visuo-perceptual capabilities and the detection of audiovisually synchronous events, performance in the UFOV task predicted performance in the AVS task, whereas performance in the CVSTM did not.

In the UFOV task, detection performance decreased with an increasing eccentricity, *F*(2, 44) = 92.01, *p* < .001 (see Fig 1B, for accuracy values of every location). The individual differences in this task were remarkably stable as indicated by high correlations among all eccentricities. These correlations were *r*(21) = .71, *p* < .001, for eccentricities of 10 deg and 20 deg, *r*(21) = .52, *p* = .011, for eccentricities of 10 deg and 30 deg, and *r*(21) = .87, *p* < .001, for eccentricities of 20 deg and 30 deg. In other words, participants who performed more accurate for peripheral probes (thus showing a larger UFOV in the original interpretation of the task) also perform more accurate for probes at the innermost circle (thus showing enhanced detection in general). For the analysis of the individual differences across tasks, we averaged accuracy across all eccentricities as a proxy for visuo-perceptual capabilities (*M* = 64.02%, *SD* = 15.56%). An analysis of the distribution of the errors across the radial axes relative to the target location (i.e. on the opposing axis, or one, or two, or three position clockwise or counter-clockwise to the target) showed that they were unsystematically distributed across all axis with values ranging from a minimum of *M* = 4.02% (*SD* = 3.42%) at a deviation of two axes in the clockwise direction to a maximum of *M* = 6.20% (*SD* = 3.20%) at a deviation of three axes in the clockwise direction, *F*(6, 132) = 2.02, *p* = .067. Therefore, the distribution of the errors is consistent with the assumption that errors in the UFOV task stem from failures of detection rather than failures of (an exact) localization.

For the analysis of the CVSTM task, we first computed the working memory capacity separately for each set size and participant according to the formula of Cowan [33]

capacity=n(objects)*(p(hits)−p(falsealarms)).

The estimated capacity values (see Fig 1D) increased with the set size, *F*(2, 44) = 26.46, *p* < .001, indicating that some participants performed at ceiling in the conditions with smaller set sizes. The estimates from the set sizes of four and six items were highly correlated, *r*(21) = .80, *p* < .001. However, the correlation between the set sizes of four and eight items, *r*(21) = .31, *p* = .151, as well as the correlation between the set sizes of six and eight items, *r*(21, *p* = .115), did not reach statistical significance. This indicates that eight items clearly exceed the capacity limitations of most participants. As the proxy of working memory capacity for the joint analysis across tasks, we therefore averaged the three capacity estimates for each participant (all results remain the same when the capacity estimation from the set size of eight items is omitted from the overall capacity estimation). The average capacity was *M* = *3*.*86*, *SD* = 0.84, which is in line with previous work on this task (see Cowan, 2000).

In the AVS task, we computed the sensitivity value d’ derived from signal detection theory across all set sizes as a marker for the performance in the AVS task. The ability of the participants to discriminate between target-absent and target-present trials declined with an increasing set size, *F*(2, 44) = 43.45, *p* < .001 (see Fig 1F). The individual differences in the different set sizes were highly correlated, *r*(21) = .78, *p* < .001, for set sizes of 2 and 3 items, *r*(21) = .81, *p* < .001, for set sizes of 2 and 4 items, and *r*(21) = .91, *p* < .001, for set sizes of 3 and 4 items. For the joint analysis across the different tasks, we calculated d’ across all set sizes as a proxy for individual differences in audiovisual integration, *M* = 1.92, *SD* = 0.72.

**Fig 2:**
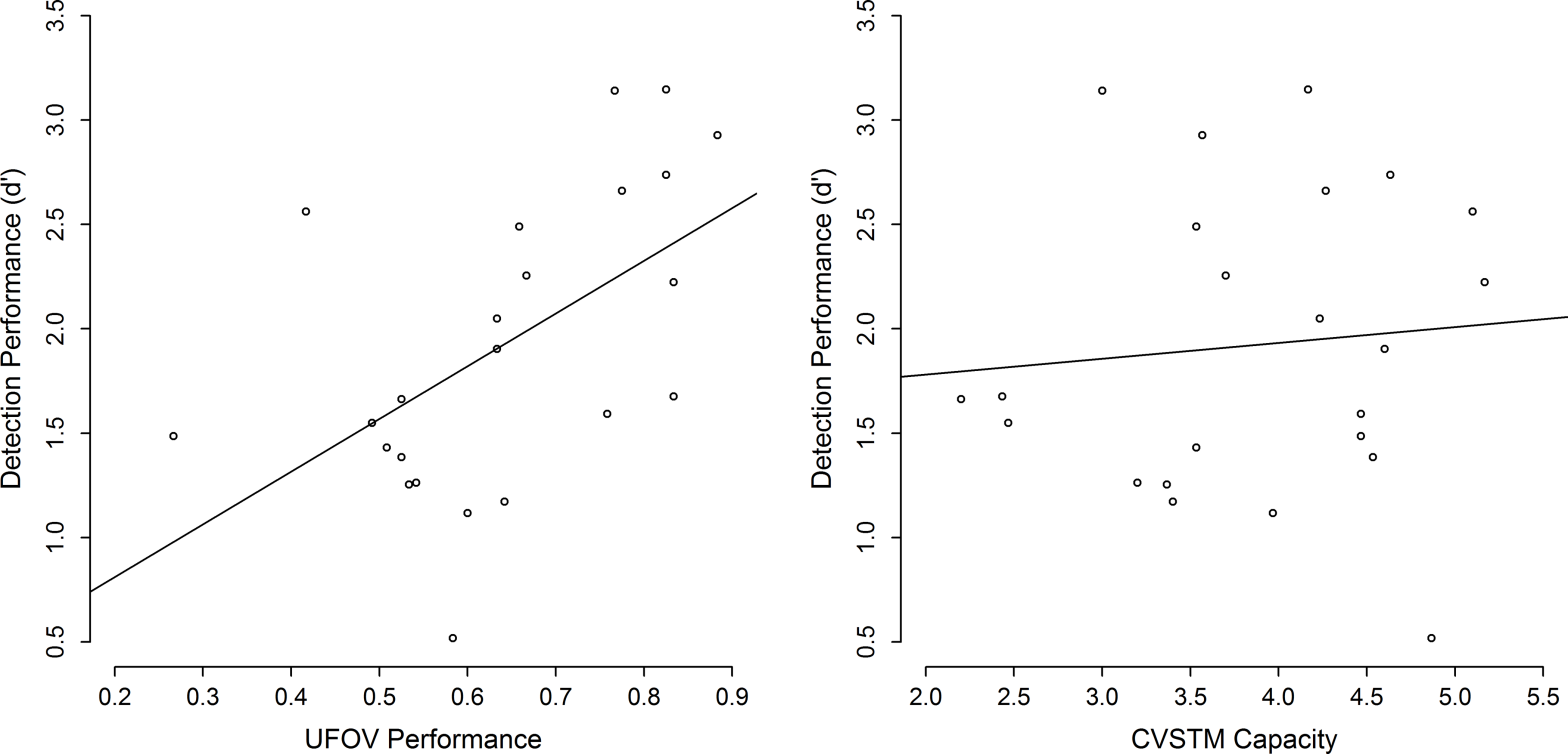
Results. Performance in the audiovisual synchrony task depending on performance in the useful-field-of-view task (left panel) as well as the color-visual-short-term-memory task (right panel). The dots indicate the observed individual performance. The solid line indicates the regression line. Performance in the useful-field-of-view task predicts performance in the audiovisual detection task.

Please note that we averaged the performance across set sizes in all task (as intended prior to the study) in order to obtain reliable individual differences for all participants. Prior to the regression analysis, we confirmed that all variables were normally distributed, all Shapiro *p*s > .366, and that both predictors (UFOV, CVSTM) were uncorrelated, *r*(21) = .03, *p* = .974. For our regression analysis (all variables were standardized), we first fitted a model that included performance in the UFOV task as well as the CVSTM task as predictors and performance in the AVS task as the dependent variable. Overall, this model successfully predicted AVS performance, *R*^2^_adj_ = .24, *F*(2, 20) = 4.44, *p* = .025. Importantly, however, within this model only UFOV performance was a significant predictor, β = 0.55, *t*(20) = 2.94, *p* = .008, whereas CVSTM performance was not, β = 0.09, *t*(20) = 0.46, *p* = .651. In our final model, we therefore included only UFOV performance as predictor. Again, UFOV performance significantly predicted AVS performance, β = 0.55, *t*(21) = 3.00, *p* = .007, *R*^2^_adj_ = .27 (see Fig 2). In order to provide an estimate of the robustness of the UFOV performance as predictor for the AVS performance, we calculated the 95%-CI [.24; .80] of β using a bootstrapping procedure with n = 10000 repetitions.

Finally, since we obtained d’-values for the AVS task, we also checked for potential correlations between the corresponding response criterion c and performance in the UFOV as well as the CVSTM task. However, we did not observe any correlations between c and the predictor variables, both *p’*s > .649.

## General discussion

The major finding of this experiment is that there is a correlation between the individual performances in the UFOV task and individual performances in the AVS task. Performance in the UFOV task predicted the success of participants in discriminating trials in which coinciding visual and auditory information was present from trials without coincidence between visual and auditory information. Importantly, this correlation cannot be attributed toward more general processes such as motivation because performance in the CVSTM task was not correlated with the other tasks. Please note that our AVS task does not allow a strict distinction between the detection of audiovisually coinciding transients and the discrimination of the prescence/absence of audiovisual coincident information. Whereas our experimental task itself requires the discrimination of trials with present/absent audiovisual coincidence, a plausible mechanism to solve this task would be to (try to) detect the presence of audiovisual coincidence and respond “absent” whenever no audiovisual coincidence is detected.

The finding that a visuo-perceptual capability such as performance in the UFOV task is related to performance in a task that requires the discrimination of audiovisually synchronous and asynchronous events might seem unintuitive at first sight. However, a careful consideration of the UFOV performance helps explaining this phenomenon. Although the UFOV task originally was designed to capture individual differences in the size of the useful field of view, the tight correlations between the individual differences across the different eccentricities indicate that the UFOV task rather captures temporal aspects of visual processing. In other words, although performance in the UFOV task declines with eccentricity, participants who perform more accurate at the outer eccentricity also perform more accurate at the inner locations. A plausible explanation for this pattern of results is that participants who seem to have a broader visual field in our experiment (as well as in previous studies reporting individual differences in UFOV performance [30,31]) just need shorter temporal intervals to detect visual stimuli within their visual field [36–38]. Given the tight connection between temporal aspects of the perception of audiovisual simultaneity and audiovisual integration [13,14], it therefore seems likely that fast and accurate visual perception strengthens effects of audio-visual integration by removing uncertainty from the visual part of the percept.

Although previous research has investigated the influence of the spatial congruency between the sources of visual and auditory information [39,40], to our knowledge no study has yet investigated the link between visuo-perceptual capabilities and the detection/discrimination of audiovisually synchronous events. Furthermore, whereas the audiovisual stimuli in most previous studies that explored the perceptual roots of audiovisual integration were isolated in the display [13,41], the visual stimulus was part of a multi-element display in our experiment. Therefore, it is straightforward that the ability to detect visual events as measured in the UFOV task also limits the detection of audiovisually coinciding transients as well as discriminating such coinciding transients from independent transients. In this regard, our results show that individuals with higher visuo-perceptual abilities can search for audiovisual synchrony more efficiently than individuals with lower visuo-perceptual abilities; see also [42].

It seems noteworthy that the spatial extent of the AVS display approximately matched the extent of the inner ring of the UFOV task. This further urges for the conclusion that it is the ability to detect briefly presented stimuli rather than the actual size of a “chronic” attentional window [25–27] that limits audio-visual integration. In fact, our observation that UFOV performance captures temporal aspects of visual processing is also relevant for the interpretation of previous studies that mainly discussed their results in terms of the spatial extend of the useful field of view [31]. Although we are certain that our study provides important insights into the perceptual roots of audio-visual integration, we (of course) acknowledge that our conclusions stem from a single correlational experiment. Thus, future research should further explore the relationship between the speed as well as the spatial extend of visual processing.

In general, our data add to the observation of large individual variance in tasks that draw upon audiovisual integration. Previous studies have already demonstrated that human observers differ in the perception of audiovisual simultaneity [41] and that these individual differences predict the relative strength of audiovisual illusions [13]. The new finding in our study is that also individual variance in visuo-perceptual processing predicts variance in the discrimination of audiovisually synchronous versus asynchronous events. In agreement with the previous studies, our experiment therefore shows that individual differences provide a powerful approach towards studying the perceptual roots of audiovisual integration.
